# Association Between Periodontal Health and Quality of Life in Patients with Psoriasis: A Cross-Sectional Study

**DOI:** 10.3390/medicina61101825

**Published:** 2025-10-12

**Authors:** Gülbahar Ustaoğlu, Şeyma Çardakcı Bahar, Ayşenur Botsalı, Özlem Saraç Atagün, Seval Ceylan Şen, Ahmet Tuğrul Su, Zeynep Hazan Yıldız

**Affiliations:** 1 Department of Periodontology, Gulhane Faculty of Dentistry, University of Health Sciences, Ankara 06010, Turkey; seyma.cardakcibahar@sbu.edu.tr (Ş.Ç.B.); ozlem.saracatagun@sbu.edu.tr (Ö.S.A.); seval.ceylansen@sbu.edu.tr (S.C.Ş.); zeynephazan.yildiz@sbu.edu.tr (Z.H.Y.); 2 Department of Dermatology and Venereology, Gulhane Training and Research Hospital, University of Health Sciences, Ankara 06010, Turkey; aysenur.botsali@sbu.edu.tr (A.B.); ahmettugrul.su@sbu.edu.tr (A.T.S.)

**Keywords:** periodontitis, psoriasis, quality of life, questionnaire

## Abstract

*Background and Objectives*: This study is aimed at evaluating periodontal health in patients with psoriasis and investigating its impact on dermatology-specific and oral health-related quality of life. *Materials and Methods*: A total of 226 individuals were enrolled, including 113 patients with clinically diagnosed psoriasis and 113 age- and gender-matched healthy controls. The periodontal parameters recorded included plaque index (PI), gingival index (GI), probing depth (PD), and clinical attachment loss (CAL). Oral health-related quality of life was assessed using the Oral Health Impact Profile-14 (OHIP-14), while dermatology-specific quality of life was evaluated with the Psoriasis Quality of Life Questionnaire (PQLQ). Psoriasis severity was measured by the Psoriasis Area and Severity Index (PASI). *Results*: Patients with psoriasis demonstrated significantly poorer periodontal parameters compared to controls, with higher PI (*p* = 0.006), PD (*p* = 0.001), and CAL (*p* = 0.041), as well as a lower number of teeth (*p* = 0.027). No significant differences in GI were observed (*p* = 0.331). Subdomain analysis of OHIP-14 indicated significantly greater functional limitation in the psoriasis group (*p* = 0.001), although no differences were detected in other domains. Positive and significant correlations were found among all the OHIP-14 subscales in both groups, and PQLQ scores were strongly correlated with OHIP-14 outcomes in the psoriasis group (*p* < 0.05). PASI scores tended to be higher among patients with periodontitis than those with gingivitis or periodontal health, but this difference did not reach statistical significance (*p* = 0.257). *Conclusions*: Psoriasis patients exhibited poorer periodontal status and reduced oral health-related quality of life compared to healthy individuals. However, differences in oral hygiene habits may also have contributed to these findings. Our findings suggest an association between psoriasis and impaired periodontal health, but due to the cross-sectional design, a causal relationship cannot be established.

## 1. Introduction

Psoriasis is a chronic, immune-mediated, inflammatory disease that primarily affects the skin and joints, with a global prevalence ranging from 2% to 3% [1,2,3]. Plaque psoriasis, the most common clinical variant, is characterized by well-demarcated, erythematous, scaly plaques and may be accompanied by nail changes and psoriatic arthritis [4]. Although not life-threatening, psoriasis significantly impairs patients’ physical, emotional, and social well-being [5]. Its pathogenesis is complex, involving both genetic predisposition and environmental triggers such as stress, infections, trauma, and lifestyle factors including smoking and alcohol consumption [6]. Immunologically, the interleukin (IL)-23/Th17/IL-17 axis plays a central role in sustaining the inflammatory response in psoriatic lesions [7].

Similarly, periodontitis is a chronic, multifactorial inflammatory disease that affects the supporting structures of the teeth. It is primarily initiated by a dysbiotic oral microbiota and results in the irreversible loss of periodontal attachment and alveolar bone [8]. Like psoriasis, periodontitis involves an exaggerated immune response and shares many of the same risk factors, including smoking, diabetes, stress, and obesity Comparable to psoriasis, periodontitis is characterized by an exaggerated immune response and shares multiple common risk factors, including smoking, diabetes, stress, and obesity [9]. Moreover, non-surgical periodontal therapy has been shown to positively influence the course of such chronic inflammatory diseases [10]. Taken together, these findings support the concept of shared pathogenic mechanisms between psoriasis and periodontitis.

Recent studies have highlighted a potential bidirectional relationship between periodontitis and psoriasis, suggesting that chronic oral inflammation may exacerbate systemic immune dysregulation [11,12]. Several observational and case–control studies have demonstrated that individuals with psoriasis exhibit more severe periodontal destruction, higher probing depths, increased clinical attachment loss, and greater tooth loss compared to healthy controls [13,14]. A recent meta-analysis of cohort and case–control studies revealed that individuals with periodontitis have a 1.55-fold increased risk of developing psoriasis [15]. Given the overlapping immunopathogenesis and comorbidity profiles of these two chronic inflammatory conditions, further investigation into their interrelationship is warranted.

The cosmetic disfigurement associated with psoriasis negatively affects quality of life (QoL), leading to psychological stress, social difficulties, and daily life limitations [16]. Patients frequently experience embarrassment, stigmatization, depression, and impaired self-esteem and body image, which can interfere with personal relationships, sexual life, sports, self-care, and professional or academic activities [17,18].

The disability associated with psoriasis has been widely investigated over the past decade using both generic and disease-specific instruments [19,20,21]. Generic measures such as the Short Form-36 (SF-36) [22], Sickness Impact Profile (SIP) [21], Nottingham Health Profile (NHP) [23], and General Health Questionnaire (GHQ) [24], as well as dermatology-specific tools including the Dermatology Life Quality Index (DLQI) [25], Skindex-29 [26], and the Psoriasis Quality of Life Questionnaire (PQLQ) [27], have been commonly applied to assess quality of life in psoriasis.

The PQLQ is used to assess quality of life, with each item offering four response options (‘nothing’, ‘some’, ‘quite’ and ‘a lot’), scored from 0 to 3, respectively. Items 6, 7, 9, and 11 addressed difficulties in daily life, and items 15, 16, and 17 focused on treatment, while the remaining questions reflected psychosocial problems associated with the disease [28].

Oral health-related quality of life (OHRQoL) is a comprehensive measure encompassing the physical, psychological, and social effects of oral health and serves as a valuable tool in assessing treatment needs and planning care. The Oral Health Impact Profile-14 (OHIP-14) is commonly employed to evaluate OHRQoL across seven subscales, reflecting different dimensions of patient experiences [29].

Although links between psoriasis and periodontitis have been reported, their impact on patient-centered outcomes remains insufficiently explored. Combining dermatology-specific (PQLQ) and oral health-related (OHIP-14) quality of life assessments with periodontal evaluation may provide a more integrated perspective.

In this study, we aimed to evaluate periodontal health in patients with psoriasis and to investigate its impact on both dermatology-specific and oral health-related quality of life, to elucidate possible shared pathological mechanisms between the two conditions.

## 2. Materials and Methods

This cross-sectional study was conducted between 20 April 2025, and 20 July 2025. The ethical approval was obtained from the University of Health Sciences Scientific Research Ethics Committee (Decision Number: 2025/163). The study was registered at ClinicalTrials.gov (ID: NCT07154420). All participants provided written informed consent in accordance with the Declaration of Helsinki before inclusion.

### 2.1. Patient Selection

Participants aged 18 to 65 years, clinically diagnosed with active psoriasis, and applying to the Department of Dermatology and Venereal Diseases, Gulhane Faculty of Medicine, University of Health Sciences, were recruited into the test group (*n* = 113). Systemically healthy participants were involved in this study from the Health Sciences University, Gulhane Faculty of Dentistry, Department of Periodontology, and matched according to age and gender to the study group patients (*n* = 113). Inclusion criteria for both groups required at least 12 teeth (excluding third molars) and the absence of contraindications for periodontal clinical examination. Exclusion criteria included other dermatological conditions, pregnancy or lactation, antibiotic use within the last 3 months, continuous use of anti-inflammatory drugs, chemotherapy or radiotherapy within the last year, or periodontal treatment within the last 6 months.

### 2.2. Dermatological Assessment

The severity of psoriasis was assessed using the Psoriasis Area and Severity Index (PASI). The PASI evaluates erythema, induration, and scaling in four anatomical regions (head/neck, trunk, upper limbs, and lower limbs), each weighted by its relative body surface area. The total PASI score ranges from 0 to 72, with higher scores indicating more severe disease [30]. Patients were further classified according to accepted thresholds as mild (PASI < 10), moderate (PASI 10–20), and severe psoriasis (PASI > 20).

In addition, dermatology-specific quality of life was assessed using the Psoriasis Quality of Life Questionnaire (PQLQ) [31]. This scale measures functional limitation, physical discomfort, psychological distress, social maladjustment, and disability. Each item was scored on a Likert-type scale, with higher scores reflecting a greater impact on daily life. Cronbach’s alpha coefficients for the subscales were calculated to confirm internal consistency.

Demographic and clinical variables, including disease duration, age at diagnosis, family history of psoriasis, smoking/alcohol consumption, and the affected body regions, were also recorded.

### 2.3. Clinical Examination

All clinical parameters were evaluated by a single experienced periodontist (Z.H.Y.), and a calibration exercise was performed to obtain acceptable interexaminer reproducibility. Periodontal examinations were performed with a Williams probe (Hu-Friedy, Chicago, IL, USA). To evaluate periodontal condition, clinical parameters including the gingival index (GI) [32], plaque index (PI) [33], probing depth (PD), and clinical attachment loss (CAL) were recorded. PD and CAL were measured at six sites per tooth and recorded to the nearest millimeter. PD was defined as the distance from the gingival margin to the base of the pocket, while CAL was the distance from the pocket base to the cementoenamel junction. Mean PD and CAL values were calculated by dividing the total scores by the number of teeth examined. The periodontal status of individuals was categorized as healthy, gingivitis, or periodontitis.

### 2.4. Data Collection

Sociodemographic and clinical data, including each patient’s age, gender, marital status, smoking habits, alcohol consumption, education level, toothbrushing frequency, and the total number of teeth, were recorded.

The Turkish version of the OHIP-14 questionnaire was administered through face-to-face interviews by a single investigator (G.U.), who was blinded to the periodontal status of the participants. The OHIP-14 consists of seven subscales, each including two items: functional limitation, physical pain, psychological discomfort, physical disability, psychological disability, social disability, and handicap. Participants rated the frequency of negative experiences on a 5-point Likert scale (0 = never, 1 = rarely, 2 = occasionally, 3 = quite often, 4 = very often). The total score ranges from 0 to 56, with higher scores reflecting a greater negative impact on oral health-related quality of life [34].

### 2.5. Statistical Analysis

The sample size was calculated using the G*Power 3.1.9.2 program [35]. Based on a similar prior study, standard deviation and effect size values were used [36]. At a 95% confidence interval (α = 0.05) with 90% power, the minimum sample size per group was calculated as 113.

Statistical analyses were conducted with SPSS (IBM SPSS Statistics, Version 26.0). Normality of the continuous variables was tested using the Kolmogorov–Smirnov and Skewness–Kurtosis statistics. Since the data showed normal distribution, parametric tests were applied. Descriptive statistics were expressed as mean ± standard deviation and frequency (%). Reliability of the scales was assessed with Cronbach’s alpha. Group comparisons were performed using the independent samples *t*-test, one-way ANOVA, and Kruskal–Wallis test, while associations between categorical variables were analyzed with the chi-square test. Pearson correlation coefficients were calculated to examine relationships among continuous variables. Statistical significance was set at *p* < 0.05.

## 3. Results

A comparative analysis of patients with psoriasis (*n* = 113) and healthy controls (*n* = 113) revealed that individuals with psoriasis exhibited significantly lower mean values for functional limitation (*p* = 0.001), while no statistically significant differences were detected for the other variables (Table 1).

The periodontal clinical parameters of the participants indicated that the PI (*p* = 0.006), PD (*p* = 0.001), and CAL (*p* = 0.041) values were significantly higher in patients with psoriasis compared to healthy controls, whereas the number of teeth was significantly lower (*p* = 0.027). No significant difference was found for GI (*p* = 0.331), although the mean value tended to be higher in the psoriasis group, suggesting generally poorer periodontal conditions in these patients (Table 2).

The total study population comprised 226 individuals, including 111 females and 115 males, with mean ages of 42.94 ± 10.22 years in the control group and 43.12 ± 11.48 years in the psoriasis group. No significant intergroup differences were observed with respect to gender (*p* = 0.894), marital status (*p* = 0.371), smoking (*p* = 0.263), alcohol consumption (*p* = 0.515), or periodontal status (*p* = 0.076). However, significant differences were identified in educational level (*p* = 0.001) and toothbrushing frequency (*p* = 0.003) (Table 3).

Pearson correlation analysis conducted among participants in the control group demonstrated that functional limitation, physical pain, psychological discomfort, physical disability, psychological disability, social disability, handicap, and the OHIP-14 score were all positively and significantly correlated (*p* < 0.05), indicating strong linear associations among these variables (Table 4).

Similarly, in the psoriasis group, significant positive correlations (*p* < 0.05) were observed between the PQLQ score, functional limitation, physical pain, psychological discomfort, physical disability, psychological disability, social disability, handicap, and the OHIP-14 score (Table 5).

Finally, the mean PASI score was highest among patients with periodontitis (3.05 ± 4.01), followed by those with gingivitis (2.36 ± 4.24), and healthy individuals (1.58 ± 3.49); however, these differences among periodontal status groups did not reach statistical significance (*p* = 0.257) (Table 6).

## 4. Discussion

Psoriasis is a chronic inflammatory skin disorder that can significantly impact the psychological well-being, occupational functioning, and overall quality of life of affected individuals [37]. Understanding the risk factors and indicators associated with the onset and progression of psoriasis is crucial. In particular, investigating the potential role of other inflammatory conditions, such as periodontitis, in the pathogenesis of psoriasis is very important. In this context, we aimed to evaluate the periodontal status of individuals with psoriasis and to explore the relationship between oral health and quality of life.

Zhang et al. [37], in their systematic review, reported a positive association between periodontitis and psoriasis, suggesting that individuals with periodontitis may have an increased risk of developing psoriasis, although a causal relationship has not been established. Similarly, in the systematic review by Ungprasert et al. [38], periodontitis was found to significantly increase the risk of psoriasis, with a pooled risk ratio of 1.55 based on two cohort and three case–control studies. Marruganti et al. [39] demonstrated in their experimental study that periodontitis and psoriasis may mutually aggravate each other, with the coexistence of both conditions leading to more severe local and systemic inflammatory responses, thus supporting a bidirectional link between the two diseases. Han et al. [40] revealed that periodontitis significantly increases the risk of developing psoriasis, with smokers experiencing an even higher risk, highlighting the synergistic effect of smoking and periodontitis in psoriasis pathogenesis. A case–control study by Mendes et al. [36] revealed that individuals with psoriasis had a higher prevalence of periodontitis compared to healthy controls, and this association strengthened with increasing severity of psoriasis; however, in multivariate analysis, factors such as number of teeth, smoking, and BMI were more strongly associated with periodontitis than psoriasis itself. A nationwide Danish cohort study found that individuals with psoriasis, particularly those with severe psoriasis and psoriatic arthritis, had a significantly higher risk of developing periodontitis, suggesting a strong association between the two chronic inflammatory conditions [41]. A case–control study conducted in a North Indian population revealed a significant association between psoriasis and periodontitis, with psoriasis patients exhibiting higher periodontal indices and more severe stages of periodontitis compared to healthy controls [42]. In our study, a higher proportion of periodontally healthy individuals was observed in the randomly selected control group compared to the psoriasis group. Patients with psoriasis exhibited significantly higher values of plaque index (PI), probing pocket depth (PPD), and clinical attachment level (CAL) compared to controls (*p* < 0.05), indicating poorer periodontal status in this population. These findings are consistent with previously reported systematic reviews and experimental studies, which have suggested an increased prevalence of periodontitis among individuals with psoriasis and a potential bidirectional relationship between the two conditions. Our findings indicate that maintaining periodontal health is particularly important in patients with psoriasis, as they exhibited significantly poorer clinical periodontal parameters compared with controls, indicating worse overall periodontal status in this population.

Various studies have been conducted to investigate the pathogenesis of both psoriasis and periodontitis, particularly focusing on their potential immunological and microbial links [43,44]. One proposed mechanism highlights the role of immune activation triggered by dysbiotic pathogens, especially Porphyromonas gingivalis. This bacterium is known to stimulate both innate and adaptive immune responses, notably through the complement system with the generation of C5a, a potent neutrophil chemoattractant. In both periodontitis and psoriasis, the inflammatory response is predominantly neutrophilic, and elevated levels of C5a have been observed in affected gingival tissues and psoriatic lesions [43]. These findings suggest that innate immune activation initiated by dysbiotic microbiota, particularly in genetically susceptible individuals, may be a common pathogenic pathway in both conditions. Emerging evidence indicates a bidirectional link between psoriasis and chronic periodontitis, both driven by dysregulated immune responses and elevated proinflammatory cytokines such as TNF-α and IL-17 [44]. Majchrzycka et al. [45] reported that psoriatic patients had significantly poorer periodontal health and higher inflammatory marker levels (CRP, IL-1α, IL-17) compared to healthy controls, suggesting a link between oral health and psoriasis severity. When examining studies that investigate the relationship between the two diseases from a genetic perspective, no significant association has been identified. Baurecht et al. [46], using the Mendelian randomization (MR) technique, found no evidence supporting a causal relationship in either direction between periodontitis and psoriasis, suggesting that the observed association in previous studies may be due to shared risk factors or confounding variables rather than a direct effect. Chen et al. [47] reported that IFIH1 plays a crucial role in linking genetic susceptibility and viral infections across multiple immune-related diseases, including psoriasis and chronic periodontitis, indicating shared molecular mechanisms underlying these conditions. Although our study did not include microbiological, biochemical, or genetic assessments, we observed that patients with psoriasis had comparatively poorer clinical periodontal parameters than the control group, including higher plaque index, probing pocket depth, and clinical attachment loss. The number of remaining teeth was also lower in the psoriasis group. These findings are consistent with the proposed pathogenic links between psoriasis and periodontitis, suggesting that dysregulated immune responses and heightened inflammatory activity may contribute to both conditions. While inflammatory mediators were not directly measured, the observed periodontal deterioration among psoriatic patients supports the potential bidirectional relationship indicated in the previous literature. These results underscore the importance of regular periodontal evaluation and tailored oral health interventions in this population.

Anxiety and mood disorders, particularly depression, are significantly more prevalent in individuals with psoriasis (up to 62%) compared to the general population (4–10%) [48]; this increased prevalence is thought to be related to the physical symptoms and appearance-related effects of the disease, as well as potential common biological mechanisms involving inflammation between psoriatic and psychiatric conditions [49]. Psoriasis affecting visible areas of the body, such as the face and hands, may increase emotional sensitivity and psychosocial burden, further impacting patients’ quality of life [50,51]. Therefore, in our study, we aimed to assess the quality of life in individuals with psoriasis using the OHIP questionnaire to better understand the psychosocial burden of the disease. Moreover, this study is the first to compare PQLQ scores with OHIP scores in patients with psoriasis. Costa et al. [52] found that periodontitis was more common in patients with psoriatic arthritis and psoriasis, and that its presence significantly worsened oral health-related quality of life (OHRQoL), especially in those with both conditions. Mishra et al. [53] revealed that individuals with concurrent periodontitis and psoriatic arthritis experienced the greatest impairment in OHRQoL, suggesting a possible synergistic effect of the two conditions on patient well-being. Another study evaluating OHRQoL reported a significant association between psoriasis and periodontitis, showing that individuals with both conditions experienced markedly worse quality of life, which was further exacerbated by the severity of each disease [54]. In our study, the OHIP scores in the psoriasis group were higher than in the control group, although no significant difference was observed in the overall total score. Subdomain analysis revealed that functional limitation was significantly higher in patients with psoriasis (*p* = 0.001), indicating greater impairment in daily activities and overall functionality. Importantly, PQLQ scores were positively correlated with the OHIP total score and all OHIP subdomains, highlighting a strong association between dermatology-specific and oral health-related quality of life measures. This consistent correlation suggests that the psychosocial burden of psoriasis extends beyond dermatological symptoms and directly influences oral health-related quality of life. These findings are in line with previous studies indicating that psoriasis and periodontitis may synergistically impair patients’ oral health-related quality of life [53,54]. Taken together, our results emphasize the importance of integrating periodontal care into the comprehensive management of psoriasis to improve both physical and psychosocial outcomes.

In our study, significant differences in toothbrushing frequency and educational level were observed between patients with psoriasis and healthy controls, which may partly explain the poorer periodontal outcomes in the psoriasis group. Similar associations have been reported in previous studies. Woeste et al. highlighted that infrequent toothbrushing (≤1 time/day) was significantly associated with tooth loss in psoriasis patients, and education level was a determinant of periodontal status [55]. Skudutyte-Rysstad et al. also emphasized that socioeconomic background and dental attendance habits could act as important confounders, although the association between psoriasis and periodontitis persisted after adjusting for these variables [13]. More recently, Polineni et al. confirmed that psoriasis and psoriatic arthritis were independently associated with periodontal inflammation even after controlling for confounders including education and toothbrushing frequency [56].Consistent with our findings, Kiernan et al. reported that patients with severe inflammatory dermatologic and rheumatologic diseases demonstrated poorer oral hygiene practices, with a lower proportion brushing twice daily, and although educational attainment did not differ significantly between cases and controls, reduced toothbrushing frequency combined with disease-related factors contributed to poorer periodontal outcomes [57]. Taken together, these results suggest that while differences in oral hygiene behaviors and socioeconomic status may contribute to periodontal deterioration, psoriasis itself appears to exert an independent effect on periodontal health. Our findings support this notion, as psoriasis patients demonstrated significantly higher plaque index (PI), probing depth (PD), and clinical attachment level (CAL) values despite these potential confounding factors.

The present study has some limitations that warrant discussion. The primary limitation is its cross-sectional design, which precludes establishing causality between psoriasis and periodontitis. Thus, while the findings highlight a significant correlation, the direction of the relationship remains unclear. Due to the low prevalence of psoriasis in the general population, obtaining large sample sizes is challenging; therefore, the number of participants in our study was relatively limited. As periodontitis and psoriasis share numerous risk factors, including smoking, diabetes, obesity, and socioeconomic status, the lack of full control over these factors represents a potential source of residual confounding. Although smoking status was recorded and did not differ significantly between groups, it remains a strong risk factor for both conditions and could have influenced the outcomes. Moreover, obesity and metabolic syndrome were not evaluated, which represents an additional limitation, as these comorbidities are highly prevalent among psoriatic patients and independently associated with periodontal disease. Another limitation is that participants were recruited from a single center and the same geographic area, which may restrict the applicability of the results to broader populations. Furthermore, oral hygiene habits were based on self-reported data, which may not fully capture actual behaviors and could introduce reporting bias. Quality of life assessments were also self-reported, which may further increase the risk of recall or response bias. Additionally, the lack of biological markers, such as microbiological, biochemical, or genetic analyses, limits mechanistic insights. Finally, the disparity in education levels between groups may act as a confounding factor, potentially influencing the observed associations between periodontal health and quality of life. Clinical studies suggest that treating either psoriasis or chronic periodontitis may positively influence the other, highlighting the need for interdisciplinary management, although the exact mechanisms linking these conditions are not yet fully understood. This study makes a novel contribution by evaluating clinical periodontal status alongside both oral- and dermatology-specific quality of life measures in patients with psoriasis, offering a comprehensive patient-centered perspective. Greater multidisciplinary collaboration between dermatologists and periodontists, through mutual recognition of dermatologic and oral manifestations, could enhance patient care and contribute to improved quality of life for individuals affected by both diseases. Furthermore, recent therapeutic approaches, including laser and other dermatological treatments, can reduce the visibility of skin lesions, potentially alleviating emotional and psychosocial burden in patients with psoriasis.

## 5. Conclusions

Patients with psoriasis showed poorer clinical periodontal parameters, fewer remaining teeth, and lower oral health-related quality of life (OHIP scores) compared to healthy controls, indicating compromised oral health and psychosocial burden. However, differences in toothbrushing frequency and educational level between the groups may also have contributed to these outcomes. Therefore, our findings suggest an association between psoriasis and impaired periodontal health, but they do not allow us to conclude a direct or independent effect of psoriasis itself. Further studies controlling for oral hygiene habits and socioeconomic factors are needed to clarify this relationship. Regular periodontal evaluation and targeted oral health interventions remain essential for individuals with psoriasis to minimize additional disease burden.

## Figures and Tables

**Table 1:** Comparative analysis of variables of interest in the studied groups.

	Control (*n* = 113) Mean ± Sd	Psoriasis (*n* = 113) Mean ± Sd	*p*
Age at diagnosis		29.14 ± 11.44	
PASI		2.16 ± 3.91	
PQLQ score		13.48 ± 9.01	
Functional limitation	1.85 ± 1.75	1.04 ± 1.57	0.001 *
Physical pain	1.68 ± 1.80	1.81 ± 1.90	0.590
Psychological discomfort	1.17 ± 1.49	1.42 ± 1.77	0.240
Physical disability	1.12 ± 1.56	1.04 ± 1.49	0.663
Psychological disability	1.26 ± 1.63	1.37 ± 1.72	0.606
Social disability	1.24 ± 1.70	1.06 ± 1.54	0.412
Handicap	1.28 ± 1.53	0.96 ± 1.40	0.104
OHIP-14 score	9.60 ± 9.72	8.72 ± 8.92	0.477

PASI: Psoriasis Area Severity Index; PQLQ: Psoriasis Quality of Life Questionnaire; OHIP-14: The Oral Health Impact Profile-14; Sd: Standard Deviation, * *p* < 0.05.

**Table 2:** Periodontal clinical parameters of the individuals in the sample.

	Control (*n* = 113) Mean ± Sd	Psoriasis (*n* = 113) Mean ± Sd	*p*
GI	1.35 ± 0.43	1.41 ± 0.43	0.331
PI	1.48 ± 0.52	1.69 ± 0.62	0.006 *
PD	2.03 ± 0.68	2.37 ± 0.83	0.001 *
CAL	2.31 ± 0.91	2.56 ± 0.91	0.041 *
Total teeth number	28.92 ± 1.77	27.69 ± 3.45	0.027 *

GI: Gingival index; PI: Plaque index; PD: Pocket depth; CAL: Clinical attachment level; Sd: Standard deviation, * *p* < 0.05.

**Table 3:** Characterization of the sample.

		Control (*n* = 113)		Psoriasis (*n* = 113)		*p*
Age (Mean ± Sd)		42.94 ± 10.22		43.12 ± 11.48		0.908
Gender	Male	*n*	%	*n*	%	0.894
		58	50.4%	57	49.6%	
	Female	55	49.5%	56	50.5%	
Marital status	Unmarried	34	54.8%	28	45.2%	0.371
	Married	79	48.2%	85	51.8%	
Smoking	Yes	78	52.7%	70	47.3%	0.263
	No	35	44.9%	43	55.1%	
Alcohol consumption	Yes	26	54.2%	22	45.8%	0.515
	No	87	48.9%	91	51.1%	
Educational level	Primary	17	30.4%	39	69.6%	0.001 *
	High school	48	57.1%	36	42.9%	
	University	45	60.0%	30	40.0%	
	Postgraduate	3	27.3%	8	72.7%	
Toothbrushing frequency	Once a day	52	46.8%	59	53.2%	0.003 *
	2–3 times a day	50	63.3%	29	36.7%	
	1–3 times a week	11	30.6%	25	69.4%	
Periodontal status	Healthy	60	58.3%	43	41.7%	0.076
	Gingivitis	41	43.2%	54	56.8%	
	Periodontitis	12	42.9%	16	57.1%	

*n*: number, * *p* < 0.05.

**Table 4:** Pearson correlation among variables in the control group.

		Functional Limitation	Physical Pain	Psychological Discomfort	Physical Disability	Psychological Disability	Social Disability	Handicap
Physical pain	*r*	0.743 *						
	*p*	0.001						
Psychological discomfort	*r*	0.511 *	0.735 *					
	*p*	0.001	0.001					
Physical disability	*r*	0.503 *	0.641 *	0.812 *				
	*p*	0.001	0.001	0.001				
Psychological disability	*r*	0.538 *	0.631 *	0.771 *	0.869 *			
	*p*	0.001	0.001	0.001	0.001			
Social disability	*r*	0.492 *	0.613 *	0.724 *	0.728 *	0.752 *		
	*p*	0.001	0.001	0.001	0.001	0.001		
Handicap	*r*	0.598 *	0.665 *	0.713 *	0.669 *	0.714 *	0.746 *	
	*p*	0.001	0.001	0.001	0.001	0.001	0.001	
OHIP-14 score	*r*	0.748 *	0.853 *	0.880 *	0.873 *	0.884 *	0.849 *	0.856 *
	*p*	0.001	0.001	0.001	0.001	0.001	0.001	0.001

OHIP-14: The Oral Health Impact Profile-14; *r*: Pearson correlation coefficients; * *p* < 0.05.

**Table 5:** Pearson correlation among variables in the psoriasis group.

		PQLQ Score	Functional Limitation	Physical Pain	Psychological Discomfort	Physical Disability	Psychological Disability	Social Disability	Handicap
Functional limitation	*r*	0.327 *							
	*p*	0.001							
Physical pain	*r*	0.287 *	0.503 *						
	*p*	0.002	0.001						
Psychological discomfort	*r*	0.411 *	0.480 *	0531 *					
	*p*	0.001	0.001	0.001					
Physical disability	*r*	0.337 *	0.541 *	0.518 *	0.733 *				
	*p*	0.001	0.001	0.001	0.001				
Psychological disability	*r*	0.299 *	0.437 *	0.506 *	0.716 *	0.652 *			
	*p*	0.001	0.001	0.001	0.001	0.001			
Social disability	*r*	0.318 *	0.453 *	0.423 *	0.613 *	0.679 *	0.617 *		
	*p*	0.001	0.001	0.001	0.001	0.001	0.001		
Handicap	*r*	0.251 *	0.349 *	0.468 *	0.524 *	0.596 *	0.555 *	0.669 *	
	*p*	0.007	0.001	0.001	0.001	0.001	0.001	0.001	
OHIP-14 score	*r*	0.408 *	0.686 *	0.737 *	0.844 *	0.854 *	0.821 *	0.801 *	0.743 *
	*p*	0.001	0.001	0.001	0.001	0.001	0.001	0.001	0.001

OHIP-14: The Oral Health Impact Profile-14; PQLQ: Psoriasis Quality of Life Questionnaire; *r*: Pearson correlation coefficients. * *p* < 0.05.

**Table 6:** Association of periodontal status and PASI score.

	*n*	PASI Score Mean ± Sd	*p*
Healthy	43	1.58 ± 3.40	0.257
Gingivitis	54	2.36 ± 4.24	
Periodontitis	16	3.05 ± 4.01	

*n*: number, Sd: Standard deviation.

## Data Availability

Dataset available on request from the authors. The raw data supporting the conclusions of this article will be made available by the authors on request.
